# Ergosterone ameliorates RRR-induced spleen deficiency by gut microbiota-gut metabolites and P38MAPK signaling pathway

**DOI:** 10.3389/fmicb.2025.1501068

**Published:** 2025-03-05

**Authors:** Ying Liu, Haiying Bao

**Affiliations:** ^1^Edible Fungi Resources and Utilization, Ministry of Agriculture and Rural Affairs, Jilin Agricultural University, Changchun, Jilin, China; ^2^College of Traditional Chinese Medicine Materials, Jilin Agricultural University, Changchun, Jilin, China

**Keywords:** spleen deficiency, intestinal barrier, gut microbiota, gut metabolomic, P38MAPK

## Abstract

Spleen deficiency is an important immune and digestive system change. Ergosterone (ER) is bioactive steroid; however, to date, no relevant studies have explored its potential efficacy in treating spleen deficiency. The aim of the present study was to investigate the therapeutic effects and mechanism of action of ER on spleen deficiency syndrome induced by Rhei Radix et Rhizoma (RRR). RRR was used to induce the development of a spleen deficiency rat model to observe changes in body weight and pathological changes in organ tissues. Additionally, the levels of relevant immune factors and gastrointestinal hormones were measured, as well as the expression of intestinal tight junction proteins and the P38MAPK signaling pathway. Changes in intestinal microbiota and metabolites were measured, and the effect of ER on the RRR-induced spleen deficiency rat model was evaluated. ER notably alleviated the symptoms of RRR-induced spleen deficiency induced in rats and offered protection against organ damage. Ergosterone can increase the expression of immunoglobulins, inhibits the increase in inflammatory factors, improve gastrointestinal hormone disorders, protect the intestinal mucosa, and repair intestinal barrier damage. The ER-treated group exhibited substantial upregulation of claudin and occludin mRNA and protein expression levels in the colonic tissue. Additionally, ER inhibited the P38MAPKsignaling pathway, thereby improving RRR induced spleen deficiency syndrome in rats. ER also influences the metabolic pathways of protein digestion and absorption, biosynthesis of unsaturated fatty acids, and arachidonic acid metabolism. In addition, ER can regulate and enhance the composition of intestinal flora in rats with spleen deficiency, increase the diversity of dominant flora, and inhibit the proliferation of harmful bacteria. ER can treat spleen deficiency syndrome by enhancing immune function, improving gastrointestinal function, repairing the intestinal barrier, and regulating intestinal flora and intestinal metabolites.

## 1 Introduction

Spleen deficiency is a common clinical syndrome characterized by symptoms, such as loose stool, loss of appetite, and mental fatigue, which are a combination of declining functions, including gastrointestinal digestion, nutrient absorption, energy metabolism, and the immune system (Zheng et al., 2020; Wang et al., 2020). The number of diseases caused by spleen deficiency has increased significantly over the past few decades. The drugs commonly used to treat diarrhea include ondansetron, rifaximin, and montmorillonite. However, these medications may result in different levels of side effects at varying degrees, including sleepiness, lightheadedness, bowel issues, and abdominal distention (Fejzo et al., 2019; Xu et al., 2022). Therefore, understanding the mechanisms underlying the therapeutic effects of drugs on spleen deficiency is crucial for developing new treatments to prevent and treat the condition. Medicinal mushrooms and their secondary metabolites such as steroidal compounds, have been identified as potential sources of natural medicines for the treatment of various diseases.

**Figure 1:**
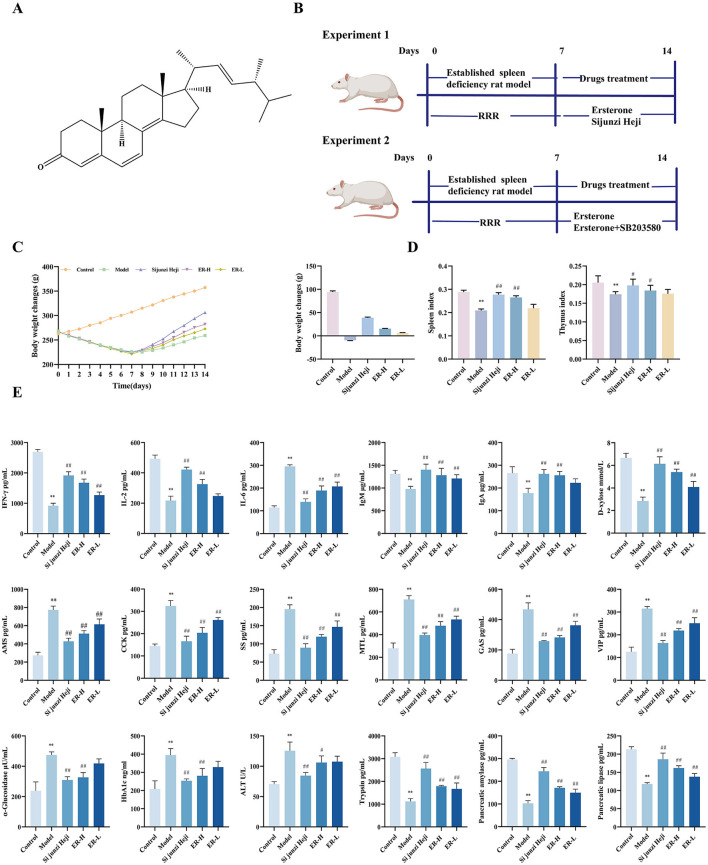
The effects of ER on spleen deficiency rat. **(A)** Ergosterone structural formula. **(B)** Schematic diagram of the animal experiment design. **(C)** Change trends and final body weight of rats during the experiment. **(D)** Spleen index and thymus index of rats. **(E)** The concentrations of IFN-γ, IL-2, IL-6, IgA, IgM, D-xylose AMS, CCK, SS, MTL, GAS, VIP, α-glucosidase, HbA1c, ATL, Trypsin, Pancreatic amylase, Pancreatic lipase (*n* = 10; ^*^P < 0.05, vs. control group; ^**^P < 0.01, vs. control group; ^#^*P* < 0.05, vs. model group; ^##^*P* < 0.01, vs. model group).

Ergosterone (ER) is a recognized bioactive steroid compound commonly present in various medicinal mushrooms, including *Tricholoma mongolicum, Gymnopilus spectabilis*, and *Inonotus hispidus*. Its structural formula is presented in Figure 1A. Modern pharmacological studies have shown that ER exhibits antitumor activity (Li et al., 2023), enhances immune function (Chen et al., 2014), and suppresses melanin and diuretic effects (Yuan et al., 2004). Nevertheless, no studies have analyzed the differences in metabolites expression and gut microbiota between spleen deficiency and ER treatment group rat. The exact roles of metabolites, gut microbiota and various physiological factors regulators involved in the invigoration of the spleen by ER remain unclear. Therefore, it is imperative to investigate the therapeutic effects of ER in spleen deficiency.

According to modern medicine, the concept of “spleen” involves the functions of multiple systems, including the digestion, endocrine and immune systems. According to Traditional Chinese medicine (TCM) the intestines is a key component of spleen function (Li et al., 2021). Recent medical research has also reported close relationships among gastrointestinal digestive dysfunction, intestinal inflammation, intestinal barrier damage, reduced immune function, and intestinal flora disturbance. The intestinal mucosal barrier protects the host from harmful substances, and disruption of the barrier can lead to increased permeability, inflammation, and other pathological changes, ultimately leading to diarrhea (Banaszak et al., 2023). The P38MAPK signaling pathway is the most important member of the MAPK family and is involved in regulating inflammatory responses. The pathway regulates immune function, cell proliferation, and apoptosis in animals and is closely related to intestinal inflammation (Lee et al., 2024). At the same time, research has found that the efficacy of treating spleen deficiency syndrome can be exerted by inhibiting the P38MAPK signaling pathway (Shi et al., 2019). Inhibiting P38MAPK signaling can reduce the levels of IL-1β, IL-8, and TNF-α in THP-1 cells and reduce damage to colon mucosal tissue (Hu et al., 2022; Li et al., 2024).

Additionally, recent research has indicated a strong connection between spleen deficiency syndrome, metabolic levels, and gut microbiota (Yan et al., 2022). Intestinal microbes and metabolites in rats with spleen deficiency changed significantly. Studies have shown that 14 types of metabolites and 15 types of intestinal microorganisms are altered in rats with spleen deficiency (Dong B. J. et al., 2024; Dong X. D. et al., 2024). Gut microbiota plays a key role in immune homeostasis. Many studies have investigated the potential role of the microbiota in regulating immunity by communicating with the immune system through the microbiome-gut immune axis (Yi et al., 2024). Gut microbiota interacts with the host primarily through metabolites, which are small molecules produced as intermediates or end-products of microbial metabolism (Xia et al., 2024). To date, gut microbiota and intestinal metabolites have been observed to be significantly altered in many immune-related inflammatory diseases. For example, some microorganisms and metabolites can improve host physiological performance, immune defense, energy metabolic processes, and intestinal mucosal barrier integrity (Zhang et al., 2020).

Based on the natural activity of ER, the present study established a rat model of RRR-induced spleen deficiency, and detected, and analyzed changes in rat organ tissues and associated biochemical parameters. This study aims to explore the role of ER in treating spleen deficiency syndrome and to clarify the specific mechanisms involved, including the regulation of P38MAPK signaling pathway, improvement of the intestinal microbiota, and maintenance of intestinal metabolites homeostasis. The results of this study will provide theoretical reference for the clinical application of ER.

## 2 Materials and methods

### 2.1 Materials

Ergosterone (lot No.: CFS202301) was purchased from Wuhan ChemFaces Biolochemical Co., Ltd (Wuhan, China). RRR was purchased from Beijing Tong Ren Tang Group Co., Ltd. (Beijing, China). Sijunzi Heji was obtained from Chongqing Tong Jun Ge Co., Ltd. (Chongqing, China). SB203580 (lot No.: 225727) was purchased from Target Molecule (Boston, MA, USA). Interleukin (IL)-2 (IL-2), IL-6, interferon-γ (IFN-γ), immunoglobulin (Ig)A, IgM, gastrin (GAS), motilin (MTL), amylase (AMS), cholecystokinin (CCK), somatostatin (SS), vasoactive intestinal peptide (VIP), d-xylose, trypsin, pancreatic amylase, pancreatic lipases, α-Glucosidase, glycosylated hemoglobin (HbA1c), and alanine aminotransferase (ALT) ELISA kits were purchased from Shanghai Jining Industrial Co., Ltd (Shanghai, China). Arachidonic acid, alpha-linolenic acid, L-arginine, L-histidine, isocitrate, 2-oxo-glutarate, L-malate, Pyruvate, 6-Keto-PGFI2and Prostaglandin I2 ELISA kits were purchased from Jiangsu Jingmei Biotechnology Co., Ltd (Jiangsu, China).

### 2.2 Experimental animals and experimental design

Six-week-old male Sprague Dawley rats, specific-pathogen-free grade, weighing 200 ± 10 g, were purchased from the Beijing Vital River Laboratory Animal Technology (Beijing, China; Certificate No.: SCXK [Jing] 2021-0011). All experimental procedures were conducted in compliance with the Regulations of the Experimental Animal Administration issued by the Ethics Committee for Laboratory Animals at Jilin Agricultural University (permit no. ECLA-JLAU-2021-0519-001).

#### 2.2.1 Experiment 1: the effect of the ER on RRR-induced spleen deficiency model rat

Fifty male Sprague Dawley rats were randomly allocated to 5 groups, each containing 10 animals, including control, model, Sijunzi Heji group, and ER groups. The ER group was further subdivided into high-dose (ER–H) and low-dose (ER-L) groups. All groups, excluding for the control group, received 1 g/mL RRR decoction administered via gavage twice daily for seven consecutive days to establish a spleen deficiency model (Zhu et al., 2022). On the 8th day, the positive control rats were administered 5.5 mL/kg of Sijunzi Heji by gavage, whereas the model group rats received an identical volume of saline intragastrically. The ER group rats received 30 mg/kg for the ER–H group and 10 mg/kg for the ER-L group via intragastric treatment. The ER dosage was determined based on a previously established method (Li et al., 2023; Li and Bao, 2023). The drug was administered for 7 days, and the body weights of the rats were recorded daily. The total experimental period was 14 days (as shown in Figure 1B).

#### 2.2.2 Experiment 2: the effect of P38 inhibitor SB203580 on the action of the ER

Sprague Dawley rats were randomly divided into 4 groups (*n* = 10), including the control group, model group (RRR 1 g/mL), RRR + ER (30 mg/kg) (RRR + ER), and RRR + ER–H (30 mg/kg) + SB203580 (10 mg/kg) group (RRR + ER–H +SB203580) groups. The rats in each group were treated as described in Section 4.2.1. The SB203580 was administered intraperitoneally at the same time as the ER–H, once a day for 7 days. The weights of the rats were recorded daily. The total experimental period was 14 days (as shown in Figure 1B).

### 2.3 Sample collection and preparation

Following the final administration, sterile EP tubes were used to collect fecal samples from rats in all experimental groups for 16s rDNA analysis. Twenty-four hours after the last administration, each group of rats were weighed accurately, and blood samples were collected from their orbits into 2 mL centrifuge tubes. The tubes were left to stand at room temperature for 30 min, after which they were centrifuged for 15 min at 3,000 rpm and 4°C. The serum was then transferred into sterile tubes and stored at −80°C for future analyses. Portions of the spleen, pancreas, thymus, colon, and liver from each rat group were fixed in 10% paraformaldehyde for histopathological assessment. Additionally, colon tissues were preserved at −80°C until metabolomic analysis was conducted.

### 2.4 ELISA analysis

As per the manufacturer's guidelines, the ELISA kit was used for analysis of the expression of various immune factors, including IFN-γ, IL-2, IL-6, IgA, IgM, gastrointestinal hormones (D-xylose, AMS, CCK, SS, MTL, GAS, and VIP) and pancreatic digestive enzymes (trypsin, pancreatic amylase, pancreatic lipase, α-glucosidase, HbA1c, and ALT), as well as the metabolites, including arachidonic acid, α-linolenic acid, L-arginine, L-histidine, isocitrate, 2-oxoglutamic acid, L-malic acid, pyruvate, 6-Keto-PGF2 and prostaglandin I2.

### 2.5 Histological analysis

Briefly, spleen, thymus, pancreas, colon, and liver tissues were collected, preserved in 10% formalin buffer, dehydrated, and embedded in paraffin. Morphological changes were assessed before hematoxylin-eosin (HE) staining (Zhong et al., 2021).

### 2.6 Western blotting

Total protein was extracted from colon tissues. Samples were washed and lysed using a buffer solution. A BCA protein assay kit (Servicebio) was used to determine the protein concentration. Proteins were separated by 10% SDS-PAGE and subsequently transferred onto PVDF membranes. After the transfer, they were blocked with 5% milk in TBS-T for 1 h, and then incubated overnight at 4°C with primary antibodies that had been diluted. After washing, the membranes were incubated with the secondary antibody at room temperature. Finally, the intensities of the blots were analyzed using AIWBwellTM (Sun et al., 2020).

### 2.7 Metabolomic analysis

A few minor modifications were made to a previously reported method for extraction and analysis of metabolites from non-volatile compounds. Freeze-dried samples were ground for 120 s at 35 Hz using a mill (MM 400; Retsch Company, Haan, Germany). Subsequently, 100 mg of the powdered sample was measured and extracted with 1 mL of 80% methanol that included 0.1 mg L^−1^ lidocaine, serving as the internal standard, at a temperature of 4°C overnight. The mixture was then centrifuged at 15,000 × g for 20 min at the same low temperature. The supernatant was collected and filtered using a filter from Millipore Corp. (Bedford, UK) before proceeding with further analyses. A Vanquish UHPLC system (Thermo Fisher Scientific, Germany) paired with an Orbitrap Q Exactive™ HF-X mass spectrometer (Thermo Fisher Scientific) was employed for the UHPLC-MS/MS analysis, which was performed at Gene Denovo Co., Ltd. (Guangzhou, China). The raw data files produced by the UHPLC-MS/MS were analyzed for peak alignment, selection, and metabolite quantification using CD3.1 software (Wu et al., 2019).

### 2.8 Gut microbiota analysis

Genomic DNA was extracted from rat fecal samples using a HiPure Stool DNA Kit (MoBio Laboratories, Carlsbad, CA, USA), according to the manufacturer's protocol. DNA quality was evaluated using a NanoDrop 2000 UV-vis spectrophotometer (Thermo Fisher Scientific) at a wavelengths of 260 nm/280 nm (Mei et al., 2022). Library sequencing was conducted using the Illumina Navoseq PE250 platform, following standard protocols provided by Gene Denovo Biotechnology Co., Ltd. Subsequently, the differences between groups were assessed using the linear discriminant analysis (LDA) effect size method, applying the Wilcoxon–Mann–Whitney test with a significance threshold of α = 0.05 and a logarithmic LDA score >3.

### 2.9 Data analysis and statistics

Data analysis was performed using SPSS software (version 20.0; IBM Corp., Armonk, NY, USA) and GraphPad Prism 7. The results are presented as means ± standard deviation and *t*-tests were used to assess inter-group variances. Multiple comparisons were conducted using one-way analysis of variance. A significance level of *P* < 0.05 indicated significant differences, whereas *P* < 0.01 indicated extremely significant differences.

## 3 Results

### 3.1 ER alleviates RRR-induced spleen deficiency in rat

Throughout the experiment, rats in the control group exhibited normal activity, shiny back hair, sensitive responses, and normal feces. After administration of RRR decoction, the rats displayed spleen deficiency symptoms, such as loose stools, decreased body weight, huddling, and laziness. The perianal area was dirty and red, and the hairs were yellowish and lacked luster, which are characteristic symptoms of spleen deficiency and consistent with the standards of the spleen deficiency rat model, suggesting successful establishment of the model (Zhang et al., 2020). The rats in the model group showed a significant decrease in weight compared to those in the control group (*P* < 0.01). After treatment, the Sijunzi Heji and ER groups showed significant improvements in clinical manifestations, with a significant increase in body weight and effective improvement of spleen deficiency symptoms (*P* < 0.01) (as shown in Figure 1C).

The organ index research findings indicated that the all organ indices in the model group decreased significantly (*P* < 0.01) compared with that in the control group. Furthermore, the Sijunzi Heji group showed significant restoration of the spleen and thymus indices of rats compared to the model group (*P* < 0.01). Additionally, the ER group demonstrated a significant increase in spleen and thymus indices in rats with spleen deficiency. Conversely, the ER–H group exhibited a significant increase in the thymus and spleen indices of spleen deficiency rats (*P* < 0.01). Thymus and spleen indices in the ER-L group showed increasing trends (as shown in Figure 1D).

### 3.2 Serum levels of cytokines, D-xylose, gastrointestinal hormones, blood glucose concentration, ATL, and pancreatic enzymes in the pancreas

Compared with the control group, the model group exhibited a substantial increase in IL-6 levels (*P* < 0.01). However, following treatment, IL-6 content in the ER group decreased significantly (*P* < 0.01), moving closer to the levels observed in the control group, resulting in a decrease in the production of inflammatory factors in the mucosa of the gastrointestinal tract. Moreover, IFN-γ, IL-2, IgA, and IgM cytokines contents were reduced markedly in the model group (*P* < 0.01). However, the ER–H group showed significant increases in IFN-γ, IL-2, IgA, and IgM contents (*P* < 0.01), whereas the ER-L group exhibited increases in IFN-γ and IgM contents (*P* < 0.01), as well as IL-2 and IgA contents (Figure 1E).

D-xylose content in the model group was considerably lower (*P* < 0.01) than that in the control group. However, D-xylose content in the ER group was significantly higher than in the model group (*P* < 0.01). This suggests that ER enhances the absorption in the small intestine (Figure 1E).

Among the six gastrointestinal hormones measured, the levels of AMS, CCK, SS, MTL, GAS, and VIP were significantly higher in the model group than in the control group (*P* < 0.01). Notably, gastrointestinal hormone concentrations in the ER dosage group were significantly elevated, suggesting effective enhancement of gastrointestinal function relative to that in the model group (*P* < 0.01). The findings demonstrate that ER ameliorates the effects of RRR decoction-induced gastrointestinal function disorders and diarrhea in rats by regulating the abnormal secretion of AMS, CCK, MTL, and GAS and significantly reducing the levels of SS and VIP (Figure 1E).

In comparison with the levels in the control group, HbA1c, and α-glucosidase were elevated significantly in the model group (*P* < 0.01), and after the administration of the drug, the ER group could significantly reduce HbA1c and α-glucosidase (*P* < 0.01), indicating that ER can reduce the blood glucose increase caused by spleen deficiency (Figure 1E).

The model group exhibited a significant increase in alanine transaminase (ALT) levels compared with that in the control group (*P* < 0.01). ER administration resulted in a reduction in ALT levels, with the most pronounced effect observed in the ER–H group (*P* < 0.05), suggesting that ER can treat abnormalities in liver function caused by spleen deficiency (Figure 1E).

Compared to the control group, the model group exhibited significantly reduced levels of trypsin, pancreatic amylase, and pancreatic lipase (*P* < 0.01). However, after treatment with ER, the ER group showed significant increases in trypsin, pancreatic amylase, and pancreatic lipase levels (*P* < 0.01). The findings indicate that ER effectively improved spleen deficiency in rats by enhancing the contents of digestive enzymes in the pancreas, facilitating the hydrolysis of dietary fat, and promoting the digestion and absorption of substances (Figure 1E).

### 3.3 Results of the HE analysis

Histological examination of the spleen revealed distinct and well-defined red and white pulps in the control group. The structure of the spleen trabeculae remained intact. In the model group, extensive necrosis of spleen cells was observed, with infiltration of inflammatory factors into large areas. The necrotic areas replaced healthy cells gradually, resulting in blurred boundaries between the white and red pulps. In the ER group, the level of damage to the spleen tissue was reduced, and there was a clear boundary between the white and red pulps. Moreover, at higher ER doses, the spleen trabecular tissue structure became more evident.

In the control group, thymocytes were arranged neatly and regularly. Cortical cells were tightly arranged, whereas medullary cells were sparsely distributed. The boundary between the cortex and medulla was distinct and the thymic corpuscles were distributed. In the model group, the thymus showed multiple necrotic cell, an unclear boundary between the cortex and medulla, and sparse arrangement of medullary cells. This indicates severe damage to the immune organ thymus in spleen deficiency model rats. Thymocytes in the ER group were better repaired, with clear boundaries between the cortex and medulla, and the thymic corpuscles were clearly visible. The improved state approached that of the control group, suggesting that the ER group has a strong ability to repair the thymus in spleen deficiency rats, reflecting the enhanced of immune activity.

In the control group, the pancreas was structurally normal, with a normal number and size of islets and an elliptical shape. In the model group, the number of pancreatic islets was reduced, the cell boundaries within the islets were indistinct, and the pancreatic alveolar structure was damaged. However, compared with the model group, the number of pancreatic islets increased to some extent in the ER group. Additionally, the structure and morphology of the pancreatic acinus recovered gradually. Histopathological sections of the pancreas showed that the morphology of the pancreatic tissue improved to different degrees in all the ER groups, particularly in the high-dose group.

In the colonic tissue, the structures of the colonic mucosa and crypts in the control group were intact and clear, with numerous evenly arranged visible goblet cells. In contrast, the model group exhibited severe damage to the colonic mucosa with a near absence of intact crypts and a diminished number of goblet cells. In the ER group, the degree of inflammatory cell infiltration and the depth of injury were reduced significantly. The number of goblet cells was significantly higher in the mucosal layer than in the model group, and the crypt structure was restored.

The liver tissue sections of the rats in control group showed radial hepatocyte structures around the central vein of the liver, and the cell nuclei were normal in morphology. The rats in the model group were loosely and irregularly arranged, with notable morphological changes. In addition, the liver sinusoids widened. The ER group exhibited improved hepatic cord morphology and structure, with nuclei returning to their normal appearance. Normal hepatic blood sinusoids were observed between the cords, and demarcation was clearly visible.

**Figure 2:**
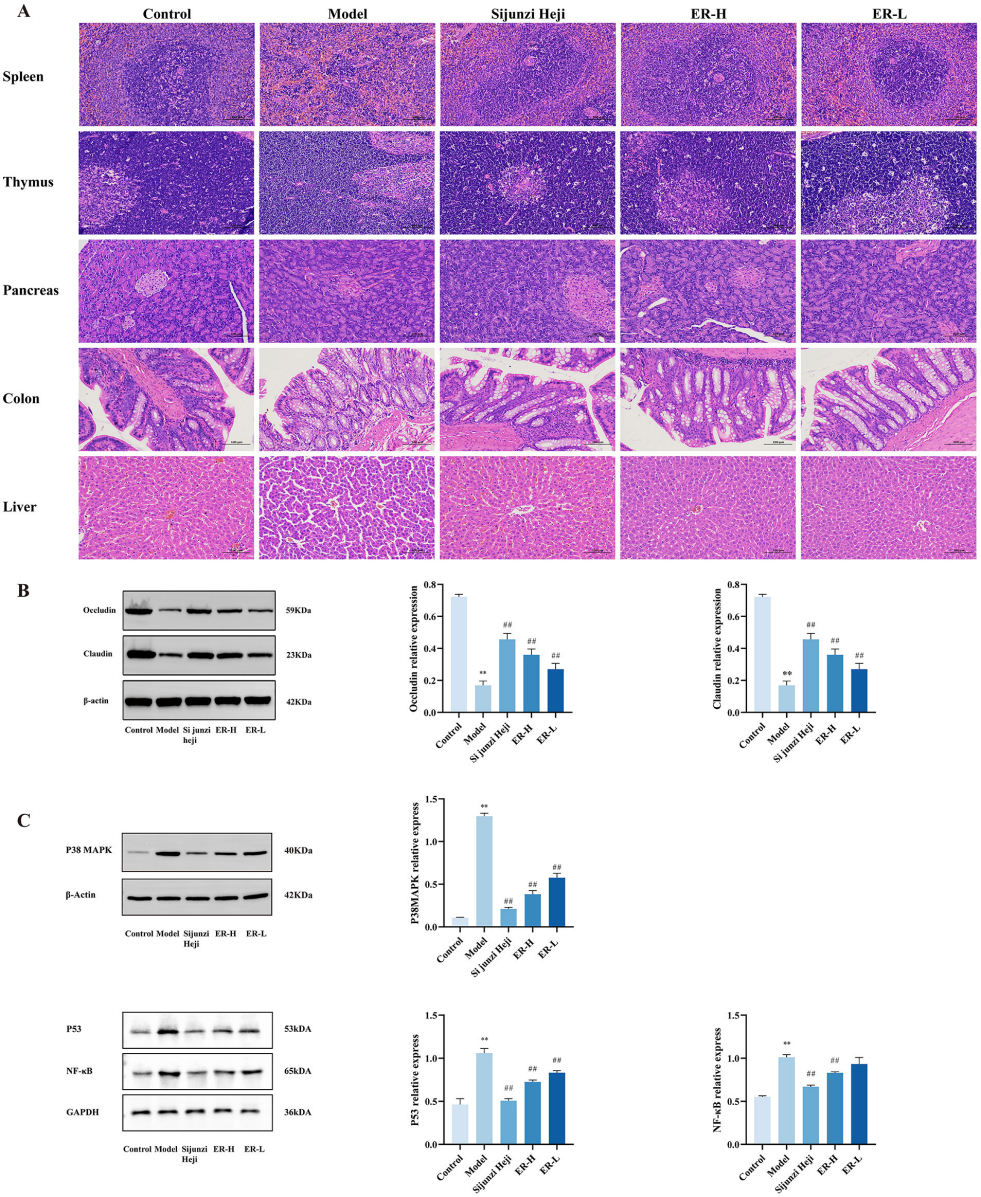
Effect of ER on the pathology and expression proteins in of spleen deficiency rats. **(A)** Pathological sections of rats with spleen deficiency, original magnification: 200× (*n* = 3). **(B)** Expression of claudin and occludin proteins in colon tissue of spleen deficiency rats. **(C)** Expression of P38MAPK, P53, and NF-κB proteins in spleen tissue of spleen deficiency rats (*n* = 3; ^*^P < 0.05, vs. control group; ^**^P < 0.01, vs. control group; ^#^*P* < 0.05, vs. model group; ^##^*P* < 0.01, vs. model group).

The spleen, thymus, immune organs, pancreas, colon, and liver of rats with spleen deficiency syndrome was damaged to varying degrees. However, after treatment with ER, significant recovery was observed (Figure 2A).

### 3.4 ER promotes the expression of occludin and claudin protein in colon tissue

To further elucidate the impact of ER on the digestive system, we investigated the expression of the intestinal tight junction proteins occludin and claudin. The model group exhibited significant reductions in the protein expression levels of both occludin and claudin (*P* < 0.01). However, after 1 week of ER treatment at doses of 30 and 20 mg/kg, there was a notable increase in protein expression (*P* < 0.01). This suggests that ER effectively modulates intestinal barrier function and enhances overall intestinal health (Figure 2B).

### 3.5 Effect of ER on P38MAPK signaling pathway in spleen tissue

The P38, P53, and NF-κB expression within the MAPK signaling pathway was assessed using western blot analysis to investigate the mechanisms behind alterations in inflammation levels. The findings indicated that RRR treatment increased the expression of P38, P53, and NF-κB protein markedly in spleen tissue, whereas its levels were reduced in a dose-dependent manner following ER administration (Figure 2C).

### 3.6 Validation of the effect of ER on spleen deficiency rats using P38 inhibitor SB203580

**Figure 3:**
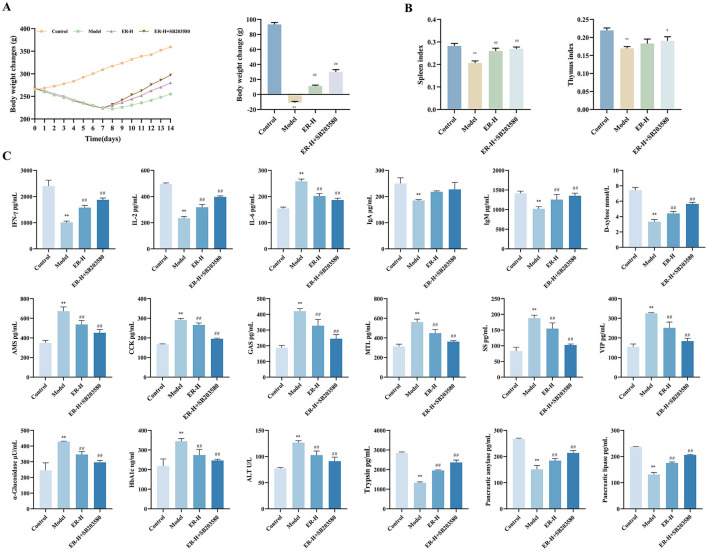
Effect of P38MAPK inhibitor on RRR-induced spleen deficiency rat. **(A)** Change trends and final body weight of rats during the experiment. **(B)** Spleen index and thymus index of rats. **(C)** The concentrations of IFN-γ, IL-2, IL-6, IgA, IgM, D-xylose AMS, CCK, SS, MTL, GAS, VIP, α-glucosidase, HbA1c, ATL, Trypsin, Pancreatic amylase, Pancreatic lipase (*n* = 10; ^*^P < 0.05, vs. control group; ^**^P < 0.01, vs. control group; ^#^*P* < 0.05, vs. model group; ^##^*P* < 0.01, vs. model group).

Given the significant inhibitory effect of ER on P38MAPK signaling *in vivo*, this pathway is considered critical in spleen deficiency rats. Therefore, the P38MAPK inhibitor SB203580, was selected for this part of the study. Compared with the model group, the body weight and organ index of rats in the pathway inhibitor group were significantly increased, which significantly enhanced the therapeutic effect of ER (Figures 3A, B).

To detect the effect of SB203580 on cytokines in rats with spleen deficiency, we detected changes in the levels of IFN-γ, IL-2, IL-6, IgA, IgM, AMS, CCK, SS, MTL, GAS, VIP, HbA1c, α-glucosidase, ALT, trypsin, pancreatic amylase, and pancreatic lipase. Compared with in the model group, the SB203580 group significantly improved the disorder of immune factors, gastrointestinal hormones, and other cytokines in rats with spleen deficiency and reinforced the therapeutic effect of the ER group on cytokines in rats with spleen deficiency (Figure 3C).

**Figure 4:**
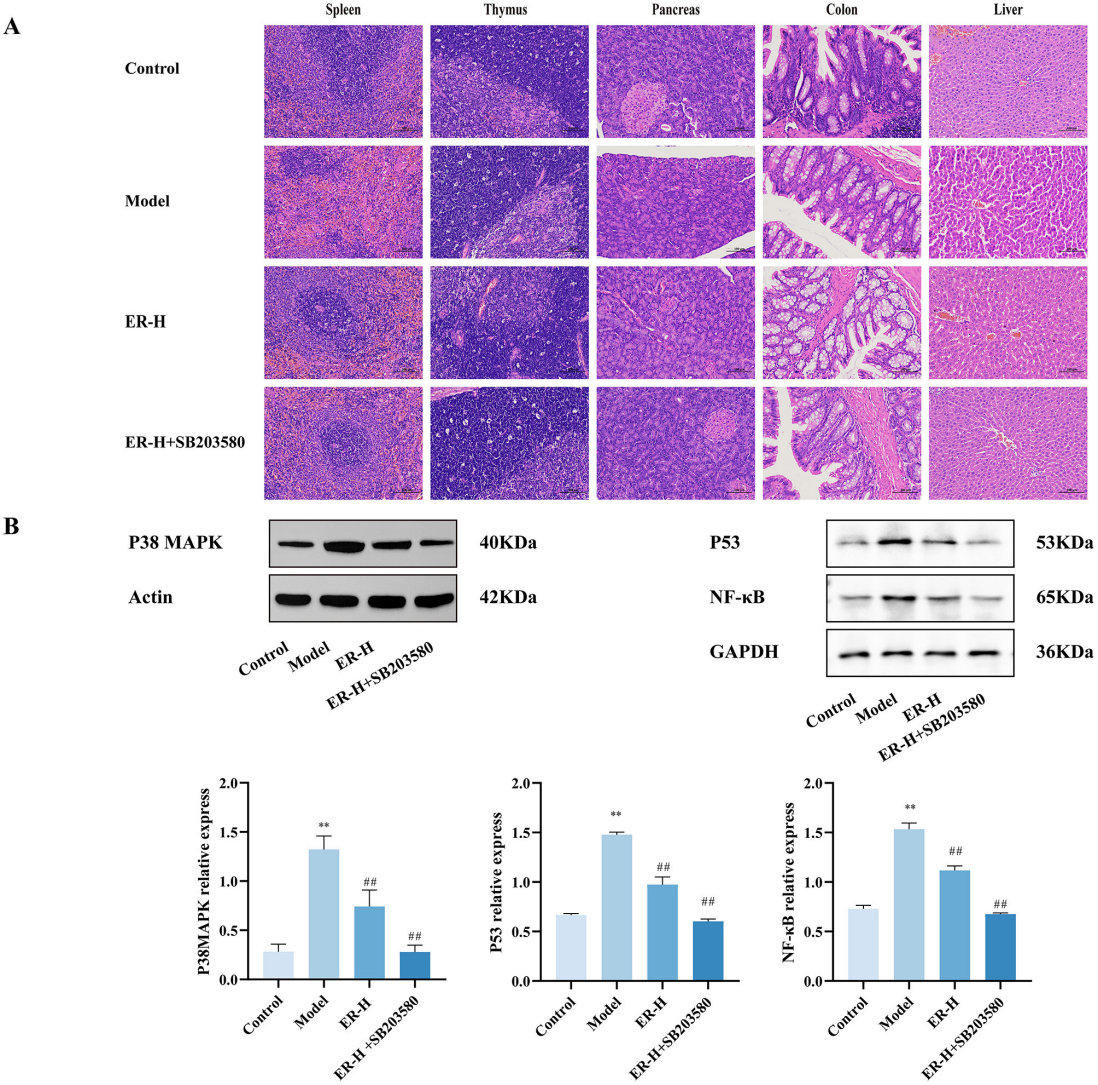
SB203580 has a synergistic effect on ER treatment of spleen deficiency rat. **(A)** Pathological sections of rats with spleen deficiency, original magnification: 200× (*n* = 3). **(B)** Expression of P38MAPK, P53 and NF-κB proteins in spleen tissue of spleen deficiency rats. (*n* = 3; ^*^P < 0.05, vs. control group; ^**^P < 0.01, vs. control group; ^#^*P* < 0.05, vs. model group; ^##^*P* < 0.01, vs. model group).

The model group suffered severe damage to the spleen, thymus, and colon, whereas the SB203580 group had minor structural damage to the spleen, thymus and colon. Compared with that in the ER–H group, the damage was significantly restored (Figure 4A).

In the model group, the protein expression levels of P38, P53, and NF-κB increased significantly (*P* < 0.01). SB203580 significantly inhibited the expression of P38, P53, and NF-κB protein in the spleen tissue of rats with spleen deficiency induced by RRR (Figure 4B).

### 3.7 Effect of ER on RRR-induced gut metabolites of rat

#### 3.7.1 Metabolites identification and quantification

**Figure 5:**
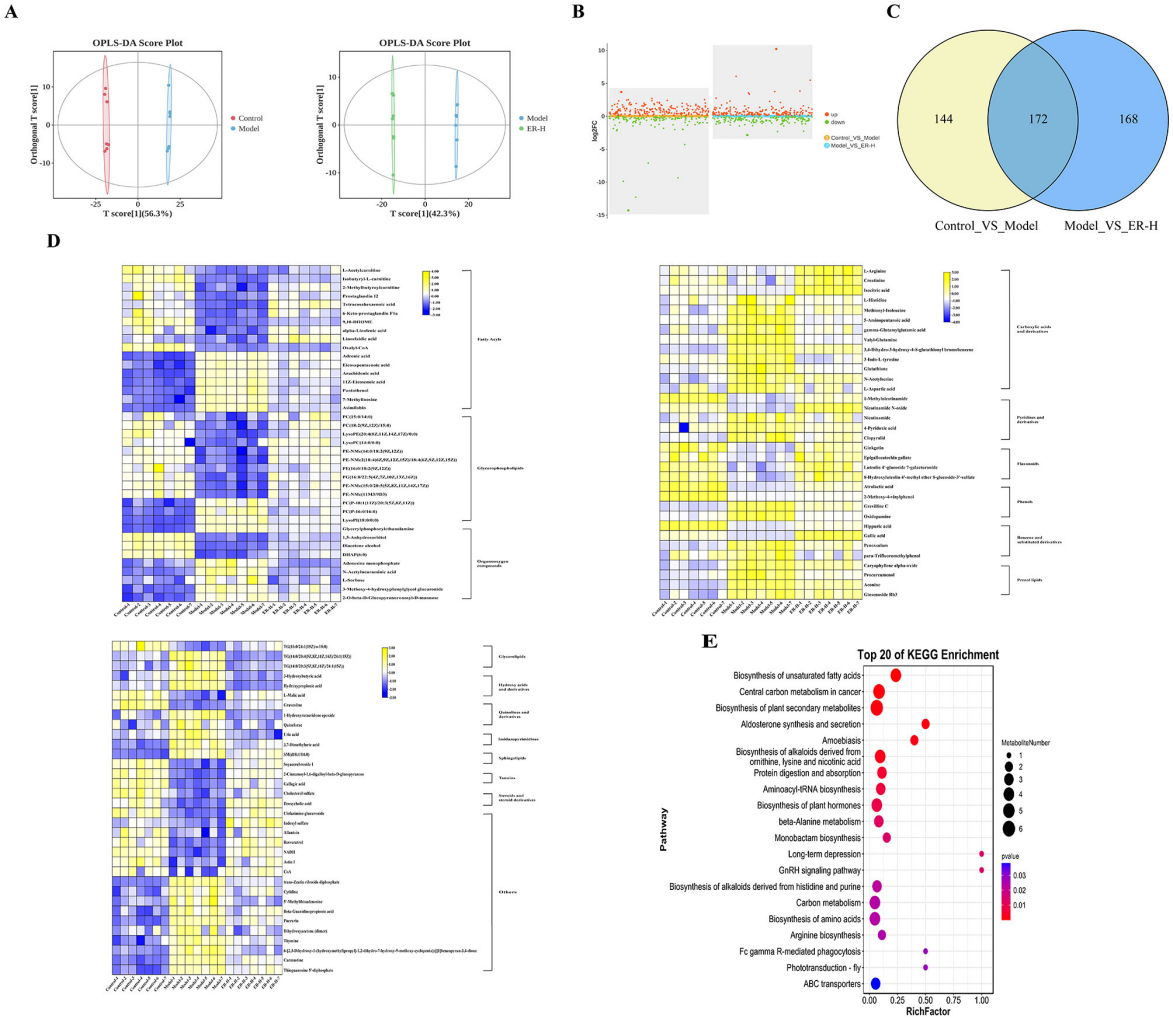
The results of metabolomics data analysis (*n* = 7). **(A)** OPLS-DA scores Plot for Colonic Metabolomics (Control_VS_Model,Model_VS_ER–H). **(B)** Colonic Metabolomics volcano plot indicating upregulated and downregulated metabolites (Control_VS_Model,Model_VS_ER–H). **(C)** Venn figure of Colonic Metabolomics (Control_VS_Model,Model_VS_ER–H). **(D)** Heat map expression of differential metabolites in Colonic Metabolomics. **(E)** Metabolic pathway of differential metabolites in Colonic Metabolomics (top 20, Model_VS_ER–H).

Research has shown that spleen deficiency syndrome is closely related to impaired gastrointestinal mucosal barrier function, and damage to colonic tissue is one of the main causes of diarrhea. To assess the effects of ER–H on physiological activities and metabolic processes in spleen deficiecy rats, colonic metabolites were analyzed using LC-MS/MS. Colonic metabolomics yielded 14,901 non-volatile metabolites. To determine the differences between the model and ER–H-treated groups, multivariate analyses were performed using a metabolic dataset. Orthogonal Partial Least Squares Discriminant Analysis (OPLS-DA) showed a clear distinction between the groups (Figures 5A, D), indicating that ER treatment caused significant changes in the metabolites.

#### 3.7.2 Differential metabolites analysis

Based on the comparison of colonic metabolites between the model group and the ER–H group 340 differential biomarkers were identified (*P* ≤ 0.05, |log2fold change| > 1, VIP > 1), of which 189 were upregulated and 151 were downregulated (Figure 5B), and out of these biomarkers, 172 biomarkers showed significant changes in metabolic levels (Figure 5C), with the levels of 107 significantly different metabolites regulated significantly by ER, and 51 and 56 significantly upregulated and 56 significantly downregulated, respectively.

#### 3.7.3 The primary differential metabolites and metabolic pathway analysis

To identify the most representative differentially expressed metabolites, 107 colonic metabolites were analyzed. The most common known classifications of differential colonic metabolites include glycerophospholipids, fatty acids, carboxylic acids, and their derivatives. The result suggests that the synthesis of metabolites of glycerophospholipids, fatty acids, carboxylic acids, and their derivatives is regulated mainly during ER–H administration to promote the normal function of material and energy metabolism and to maintain metabolic balance and homeostasis. Heat maps were plotted to visualize the differences between the 107 colonic compounds (Figure 5D) after ER–H administration.

**Figure 6:**
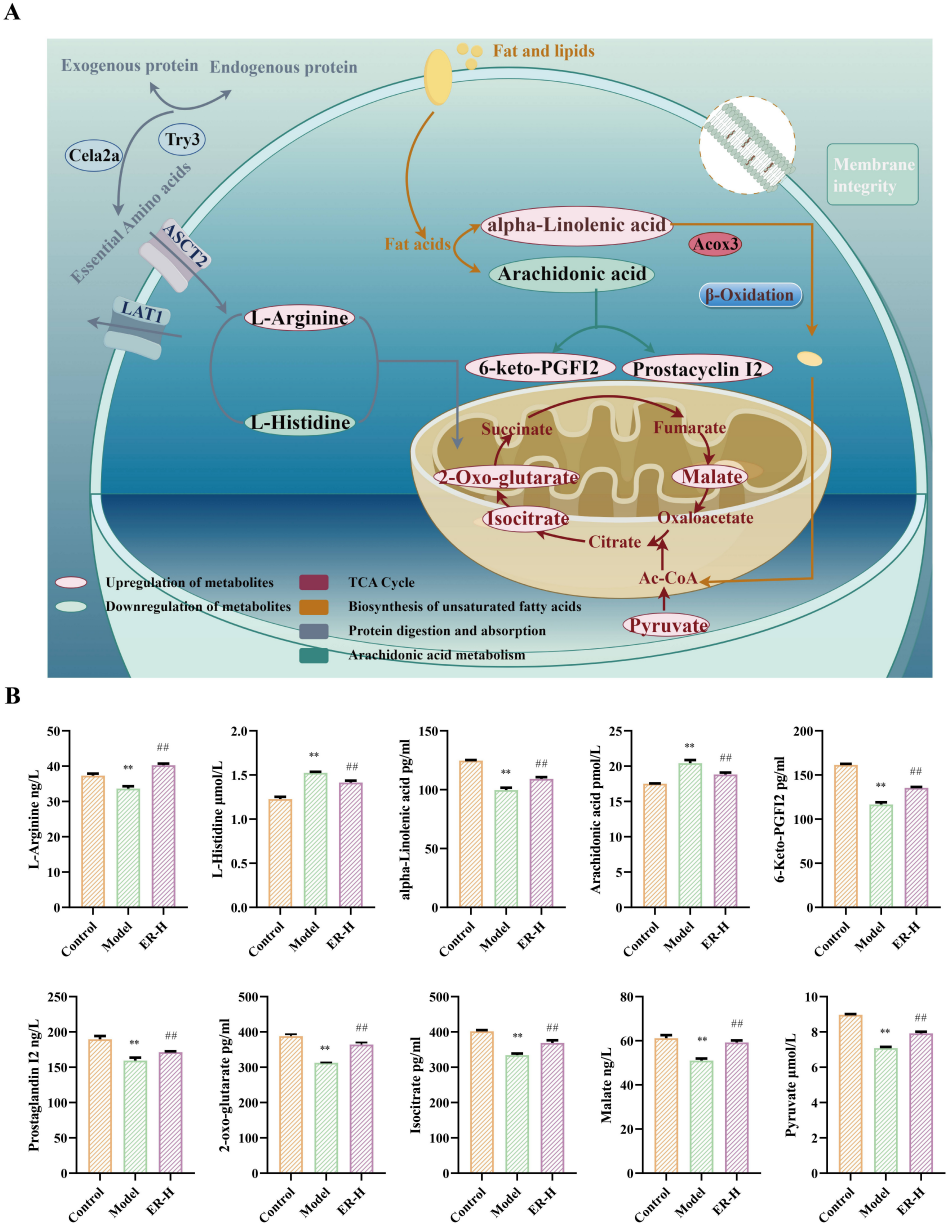
The metabolic pathways with potential markers affected by ER–H in treating spleen deficiency syndrome. **(A)** Relative abundance of key metabolites significantly affected by ER–H at the metabolic pathways. ^*^P < 0.05, vs. control group; ^**^P < 0.01, vs. control group; ^#^*P* < 0.05, vs. model group; ^##^*P* < 0.01, vs. model group. **(B)** The key metabolic pathways significantly affected by ER–H. The metabolites that are marked in *red* demonstrate a notable increase in expression levels as a result of the treatment with ER–H. In contrast, the metabolites that are identified in green show a significant decrease in their expression.

Enrichment analysis of metabolic pathways is crucial for understanding disease pathogenesis. We screened the Kyoto Encyclopedia of Genes and Genomes (KEGG) profiles of the differential metabolites to analyze the key metabolic pathways associated with the development and progression of spleen deficiency in rats. A total of 93 metabolic pathways were involved with the 107 differential colonic metabolites, and the top 20 metabolic pathways with the highest rankings were selected for further analysis (Figure 5E). The metabolites reached normal levels and metabolic pathway disorders improved after ER–H treatment in spleen deficiency rats, indicating that ER–H had significant therapeutic effects on spleen deficiency diarrheal rats. The key metabolites, arachidonic acid, alpha-linolenic acid, L-arginine, L-histidine, isocitrate, 2-oxo-glutarate, malate, pyruvate 6-Keto-PGFI2 and Prostaglandin I2, were mainly involved in the regulation (Figure 6A). The major metabolic pathways were protein digestion and absorption, biosynthesis of unsaturated fatty acids, arachidonic acid metabolism, and the TCA cycle (ranked after 20). We then conducted ELISA analysis of key differential metabolites and found that ER administration could significantly regulated metabolic disorders (Figure 6B).

### 3.8 Effect of ER on RRR-induced gut microbiota of rat

#### 3.8.1 ER regulates gut microbiota diversity

**Figure 7:**
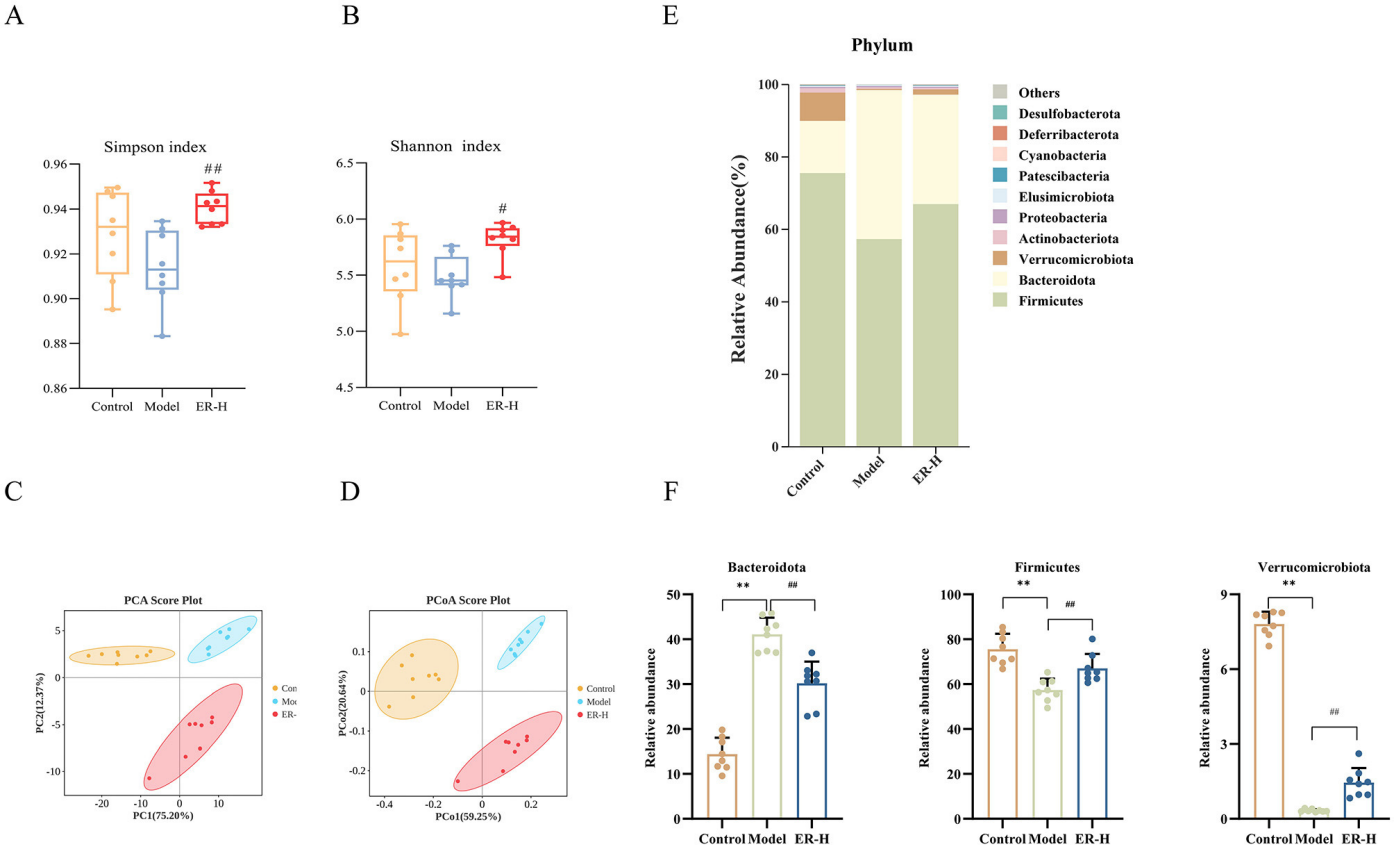
Results of gut microbiota data analysis (*n* = 8). **(A)** Rank abundance. **(B)** Shannon curve. **(C)** Simpson curve. **(D)** Analysis of gut microbiota using PCA was conducted on the OUT data from each sample across the three groups. **(E)** The relative abundance of phylum. **(F)** Species significantly influenced by ER–H at the phylum taxonomic level in spleen deficiency rats. ^*^P < 0.05, vs. control group; ^**^P < 0.01, vs. control group; ^#^*P* < 0.05, vs. model group; ^##^*P* < 0.01, vs. model group.

To confirm ER-mediated regulation of intestinal bacteria, we tested the intestinal flora in fecal samples of rats in the control, model, and ER groups. Additionally, we assessed the alpha diversity of the gut microbiota using the Shannon and Simpson indices. Our findings revealed that ER–H exhibit significant biological diversity. The α-diversity in the ER–H group was significantly higher than that in the model group, suggesting that it has an important role in maintaining the richness of intestinal flora. The ER–H group exhibited significantly greater alpha diversity than the model group, indicating its potential role in preserving the richness of the intestinal microbiota (Figures 7A, B). Overall evaluation using PCA and PCoA revealed that ER–H administration improved the intestinal flora in rats (Figures 7C, D).

#### 3.8.2 Species composition analysis of intestinal flora species

The bar chart shows the microbial community composition and abundance at the phylum and genus levels in the control, model, and ER–H groups. The 10 most abundant bacteria are shown in Figure 7E. Ten gut microbiota compositions were detected at the phylum level, with Firmicutes, Bacteroidetes, and Verrucomycetes being the dominant phylum (Figure 7F). Bacteroidetes is considered a mucosa-associated microbiota and Firmicutes a fecal-associated microbiota, dysbiosis of these microbiotas can make the intestinal mucosa more susceptible to bacterial infections and cause gastrointestinal disorders, such as diarrhea and enteritis. The relative abundance of Bacteroidetes was higher in the model group than in the control group, whereas the relative abundances of Firmicutes and Verrucomicrobia were lower. Compared to the model group, ER–H administration significantly decreased the abundance of Bacteroides and increased the abundance of Firmicutes and Verrucomicrobia. The results suggest that ER–H has an ameliorative effect on the intestinal flora at the phylum level and can ameliorate diarrhea in spleen deficiency rats.

**Figure 8:**
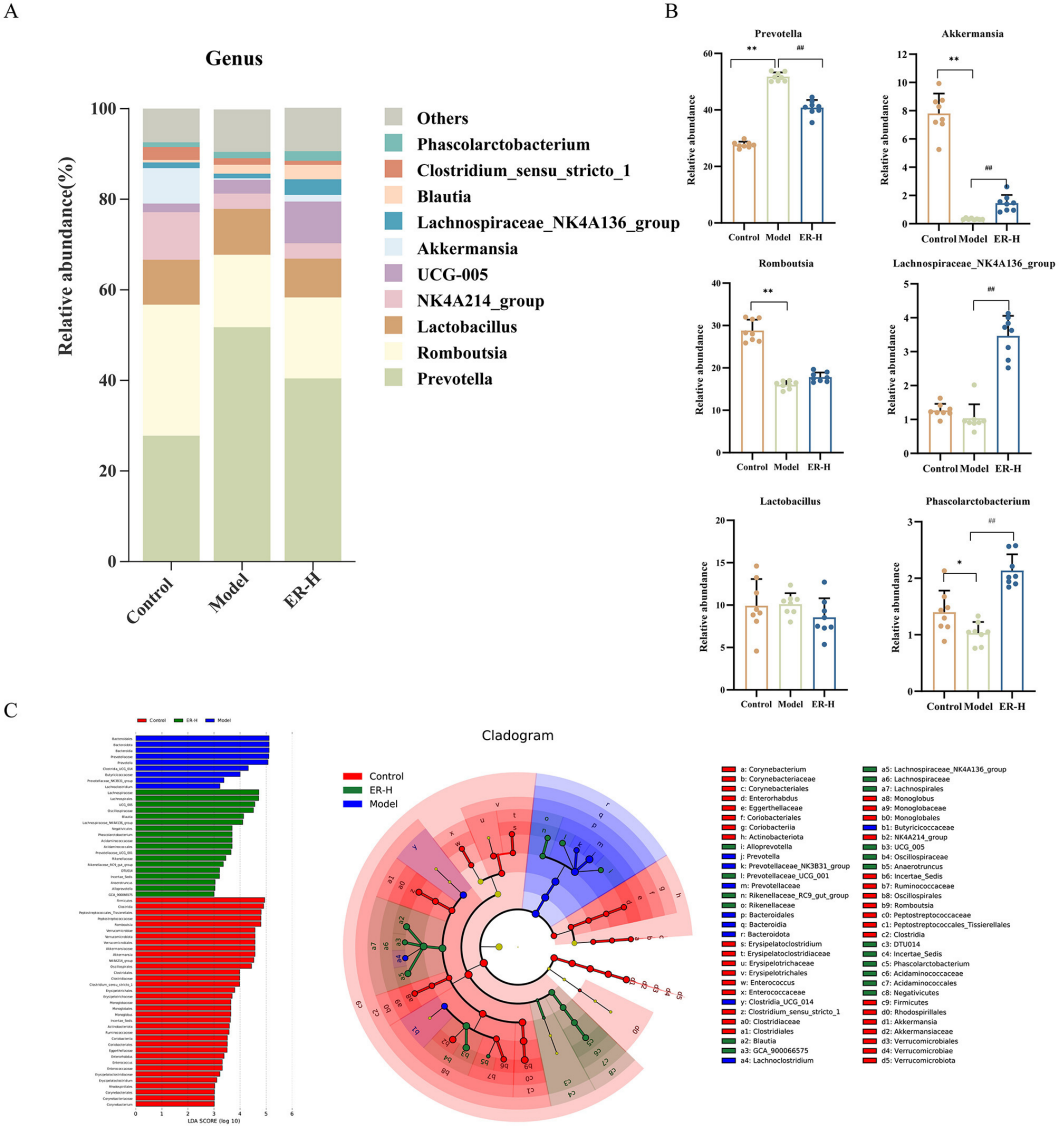
Effects of ER–H on the composition of gut microbiota at the genus level in rats with spleen deficiency. **(A)** Bar graph illustrating the relative abundance of species at the genus taxonomic level. **(B)** The relative abundance of key genus. ^*^P < 0.05, vs. control group; ^**^P < 0.01, vs. control group; ^#^*P* < 0.05, vs. model group; ^##^*P* < 0.01, vs. model group. **(C)** LEfSe analysis of dominant biomarker groups among different groups.

By comparing the composition of the gut microbiota between groups at the genus level, the top 10 significant genera (Figure 8A) were Prevotella, Romboutsia, Lactobacillus, Akkermansia, Lachnospiraceae_NK4A136_group, NK4A214_group, UCG-005, Blauta, Clostridium_sensu_stricto_1, and Phascolarctobacterium. Among them, those that played a key role in regulation were Prevotella, Romboutsia, Akkermansia, Lachnospiraceae_NK4A136_group, Phascolarctobacterium and Lactobacillus (Figure 8B). Implementation of the intervention resulted in a notable change in the relative abundance of several bacterial taxa compared to the model group. Notably, there were a significant increases in the relative abundances of Romboutsia, Akkermansia, Lachnospiraceae_NK4A136_group, and Phascolarctobacterium, whereas the relative abundances of Prevotella and Lactobacillus experienced notable decreases in the intestinal microbiota profile of rats suffering from spleen deficiency and diarrhea. It reduced the levels of Prevotella to some degree while boosting the relative abundance of beneficial probiotics such as Romboutsia, Akkermansia, and Lachnospiraceae_NK4A136_group, and improved the disorder of Lactobacillus. Consequently, ER–H is capable of restoring the intestinal microenvironment in rats with spleen deficiency while modulating the imbalance of intestinal flora.

#### 3.8.3 Significant differences between samples

Figure 8C illustrates the species that exhibited LDA scores exceeding 3 (*P* < 0.05), indicating that the difference was significant for these biomarkers. The figure shows the species with significant differences in abundance among different groups, and the length of the histogram represents the size of the significantly different species. The relative abundances of Romboutsia, Akkermansia, NKA4A214_group, Clostridium_sensu_stricto_1, Monoglobus, Incertae_Sedis, Enterorhabdus, Enterococcus, Erysipelatoclostridium, and Corynebacterium were higher in the control group. However, the model group showed higher relative abundances of Prevotellaceae, Prevotellaceae_NK3B31_group, and Lachnoclostridium. Additionally, in the ER–H group, UCG_005, Blautia, Lachnospiraceae_NK4A136_group, Phascolarctobacterium, Prevotellaceae_UCG_001, Rikenellaceae_RC9_gut_group, Anaerotruncus, Alloprevotella, and GCA_900066575 were the most abundant.

## 4 Discussion

Spleen deficiency diarrhea is a prevalent gastrointestinal syndrome in clinical practice (Wang C. X. et al., 2024; Wang K. et al., 2024; Wang X. S. et al., 2024). Many modern gastrointestinal diseases, such as gastric ulcers, irritable bowel syndrome and functional diarrhea, fall under the spleen deficiency category in TCM (Li et al., 2019) Spleen deficiency syndrome is a complex condition caused by multiple factors and characterized by clinical manifestations such as fatigue, weight loss, and diarrhea. The pathogenesis of this syndrome is primarily associated with an imbalance in intestinal flora, inflammation, abnormal secretion of gastrointestinal hormones, and damage to the intestinal barrier (Wang et al., 2022; Xie et al., 2021). Severe spleen deficiency syndrome can also induce damage to other organs and the occurrence of diseases, such as colitis (Wang C. X. et al., 2024; Wang K. et al., 2024; Wang X. S. et al., 2024), immune disorders (Zhao et al., 2023), and water metabolism and circulation disorders (Chen et al., 2023). Common drugs currently used to treat spleen deficiency exhibit side effects. As TCM compound, the main ingredients of Sijunzi Heji are Ginseng Radix et Rhizoma, Atractylodes Macrocephalae Rhizoma, Poria and Glycyrrhizae Radix et Rhizoma Praeparata Cum Melle. It had no adverse effects; therefore, we chose it as a positive control. The spleen invigorating effects of ER as a compound with multiple medicinal activities has not yet been reported. Therefore, we selected the natural product ER to treat spleen deficiency induced by RRR and study its mechanism of action.

Many rats with spleen deficiency experience diarrhea and weight loss (Dong et al., 2023). ER interventions can significantly improve such conditions and exert a therapeutic effects. In response to the low immune function condition of spleen deficiency syndrome rats, ER enhanced the immune activity of the body by increasing the contents of IgA, IgM, IFN-γ, and IL-2 in the mucosal immune system, which are key immune molecules of the body, and at the same time decreased the content of IL-6 inflammatory cytokine secretion caused by spleen deficiency. ER can treat the symptoms of spleen deficiency induced by RRR decoction in rats by regulating gastrointestinal hormones and repairing intestinal damage. Additionally, ER can increase the content of pancreatic enzymes, which help in repair pathological damage to pancreatic tissue. This finding aligns with previous conjectures, suggesting a connection between spleen deficiency and pancreatic damage. Notably, in diabetes, glycated hemoglobin and α-glucosidase are key indicators of blood sugar levels. Abnormally high values of these indicators indicate an increase in blood glucose concentration (Xue et al., 2023). The ER plays a crucial role in maintaining a stable blood sugar concentration by reducing the concentration of glycated hemoglobin and α-glucosidase, thereby preventing diabetes induced by spleen deficiency. Patients with liver injury exhibit short-term gastrointestinal symptoms such as elevated serum ALT, AST, and bilirubin levels and acute liver failure, and various serious complications can occur when the disease course is aggravated (Wang C. X. et al., 2024; Wang K. et al., 2024; Wang X. S. et al., 2024). There was a significant reduction in ALT levels and the repair of damaged liver tissue following ER administration. The intestinal epithelium is the main barrier between the body's immune system and external environment. Spleen deficiency affects colon permeability, leads to diarrhea and pathological damage. Studies have shown that the expression of claudin and occludin proteins in rats with spleen deficiency syndrome is significantly reduced (Tu et al., 2020), wherease ER treatment significantly increases the expression of claudin and occludin proteins and mRNA, indicating that ER could effectively alleviate diarrhea caused by colonic absorption disorders induced by the aqueous decoction of RRR.

Spleen deficiency can impact the expression of P38 in the MAPK signaling pathway (Deng et al., 2018). Activation of the P38MAPK signaling pathway can promote the secretion of cytokines such as IL-6 and TNF-α, causing intestinal mucosal inflammatory response. At the same time, activation of the P38 MAPK signaling pathway can inhibit the expression of ZO-1, Occludin and other proteins, leading to the destruction of the intestinal tight junction structure (Chang et al., 2020). P53 and NF-κB proteins are downstream transcription factors of P38. By inhibiting the expression of P38MAPK protein, the contents of P53 and NF-κB protein can be reduced to reduce intestinal oxidative stimulation damage and inflammatory response (Li et al., 2017; Ma et al., 2022). The consumption of ER has been shown to reduce the expression of P38, P53, and NF-κB, inhibit inflammatory factors, and enhance the expression of tight junction proteins, thereby effectively inhibiting the occurrence of inflammatory responses. The present study showed that the combined use of P38MAPK inhibitors and ER significantly enhanced and improved the decrease in body weight and organ index in rats with spleen deficiency, reduced the release of inflammatory factors, regulated gastrointestinal hormone disorders, reduced the expression of P38MAPK protein in the spleen, and enhanced immunity and digestive function, thus providing new strategies and treatment methods for spleen deficiency syndrome.

Through analysis of metabolites in the colon, we identified the relationship between ER spleen invigorating mechanism and intestinal metabolism, as well as the key regulatory substances of intestinal metabolites that are involved in the mechanism. The substances are primarily involved in protein digestion and absorption, unsaturated fatty acid metabolism, arachidonic acid metabolism, and the TCA cycle. Modern pharmacological research has indicated that such signaling pathways are associated with the genesis and progression of spleen invigoration and replenishtion qi effects (Ng et al., 2024; Zhan et al., 2024; Zhang et al., 2021). Metabolomic analyses revealed that the pathways were significantly regulated in rats following ER treatment. Such regulation results in changes in the circulation of TCA energy and ultimately alleviates the metabolic disorders caused by spleen deficiency. Protein digestion and absorption are critical for the utilization of proteins by tissues and affect amino acids metabolism. This involves amino acids and proteases that participate in the synthesis and catabolism of nutrients, with a dual function in terms of nutrition and immune regulation. The functions improve the intestinal mucosal barrier and maintain intestinal health (Yang et al., 2021). ER administration and treatment substantially increased L-arginine metabolism, thereby enhancing T cell function. It also decreases L-histidine metabolism, leading to increased α-oxo-glutarate synthesis and promotion of TCA circulation for energy production. Unsaturated fatty acids play a crucial role in maintaining normal physiological functions cells. The abnormal metabolism of unsaturated fatty acids is closely linked to inflammation, as it can activate inflammatory factors (Chen et al., 2022). Compared with the model group administration resulted in an increase in the levels of alpha-linolenic acid, whereas the levels of arachidonic acid, and 11Z-eicosenoic acid decreased. Arachidonic acid and its metabolites are disease biomarkers in the arachidonic acid metabolic pathway (Li et al., 2015). Arachidonic acid enhances inflammatory responses by recruiting and activating T cells and antigen-presenting cells. Notably, ER treatment inhibits the inflammatory response by reducing arachidonic acid content, while increasing the levels of its metabolites, prostaglandin I2 and 6-keto-prostaglandin F1a, through the action of cyclooxygenase. 6-keto-PGF1α acts as a metabolite of prostaglandin I2, both of which have the ability to dilate blood vessels and increase blood flow. This helps address the issue of insufficient blood volume caused by the reduced production of qi and blood in cases of spleen deficiency.

The composition of the intestinal flora in rats exhibiting spleen deficiency changed significantly when compared with that in the control group, based on an analysis of their intestinal microbiota. ER substantially regulates the composition and diversity of intestinal flora in spleen deficiency rats and improves spleen deficiency associated with Firmicutes, Bacteroidetes, and Verrucomycetes. Abnormal alterations in the numbers of Prevotella and Lactobacillus in the intestines of spleen deficiency rats can lead to intestinal flora disorders, and in turn, enteritis and diarrhea (Zhang et al., 2023; El-Salhy et al., 2019), which are the main causes of diarrhea in spleen deficient rats, a finding that is also consistent with our results. Probiotics have anti-inflammatory effects and can partially prevent intestinal colonization by pathogenic bacteria (Wu et al., 2020; do Carmo et al., 2018). In the present study, ER intervention substantially mediated the imbalance in Lactobacillus levels, enhanced the release of IgA, and consequently, activated B lymphocyte and T cell function. After treating rats with spleen deficiency and ER, the abundance of Akkermansia increased substantially. The increase inhibits the secretion of the inflammatory factor IL-6 and leads to higher expression of occludin and claudin, which help repair the damaged intestinal barrier. The ER can increase probiotics, inhibit pathogenic bacteria, stabilize the intestinal environment, and restore homeostasis of the mucosal immune system. It can also regulate the immune response, maintain metabolic balance, and promote the proliferation and differentiation of intestinal epithelial cells to invigorate the spleen and replenish qi. Although the present study confirmed the effects of ER on the composition and diversity of intestinal microbiota, further systematic studies are required to understand the biological mechanisms of ER.

**Figure 9:**
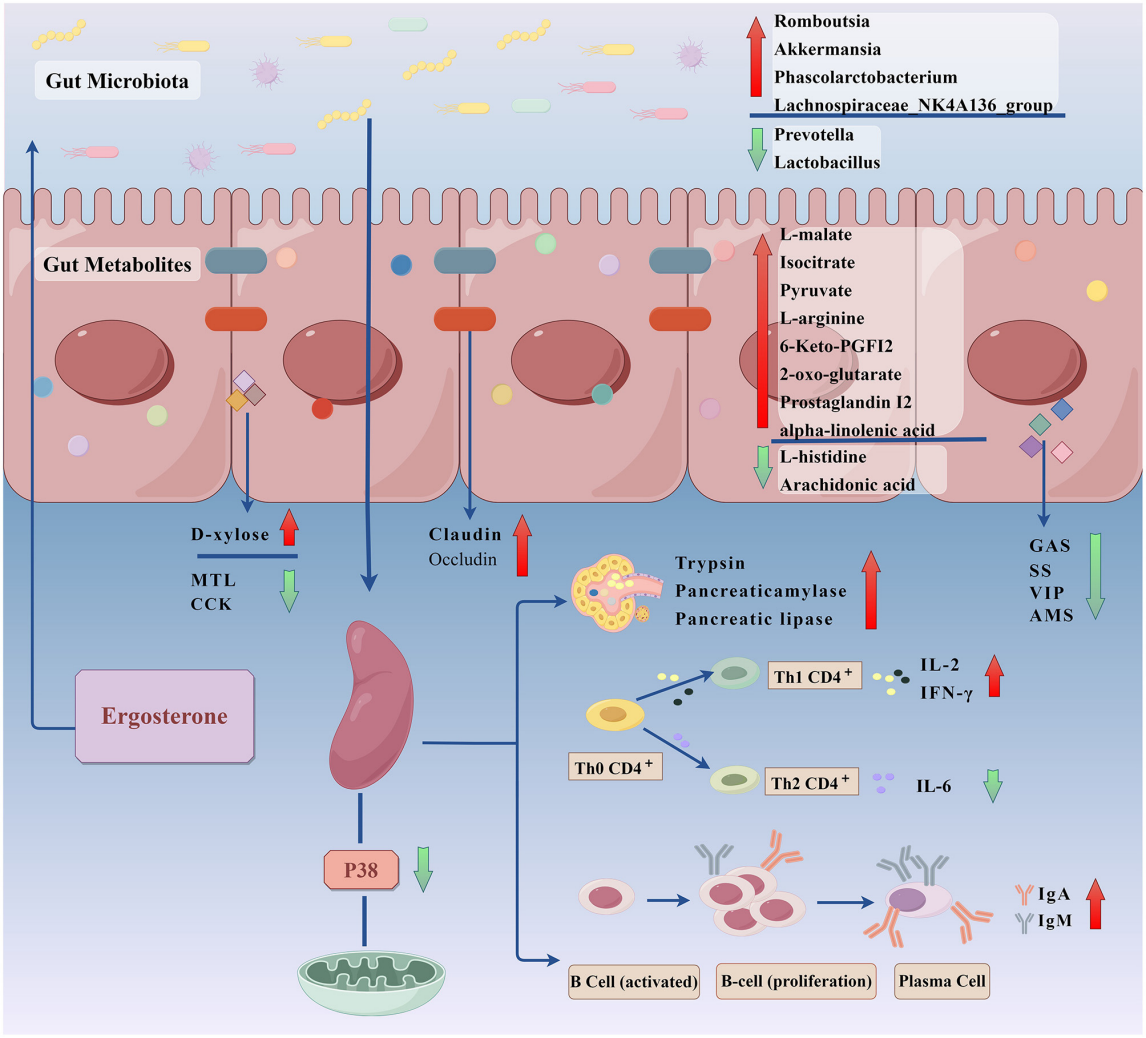
To explore the invigorating the spleen effect and mechanism of ergosterone in rats with spleen deficiency through gut microbiota-gut metabolites (By Figdraw).

In summary, the present study used RRR-induced splenic deficiency rats as an experimental model and it explored the underlying mechanisms of ER efficacy in treating spleen deficiency based on metabolomics and gut microbiota analysis. The mechanism by which the ER invigorates the spleen involves improvement of gastrointestinal absorption, regulation of gastrointestinal hormone disorders, repair of intestinal mucosal damage, and increase of the release of digestive enzyme. In addition, it can invigorate the spleen by repairing organ damage, improving humoral immune function and inhibiting the P38MAPK signaling pathway. ER treatment substantially improved intestinal endophyte disorders and metabolic abnormalities in spleen deficient rats. We intend to further explore the role of ER in treating spleen deficiency to gain a more comprehensive understanding of the mechanism of the spleen invigorating effects (Figure 9). The spleen is the largest immune organ in the human body, accounting for 1/4 of the lymphoid tissue in the body. It contains a large number of lymphocytes and it is the hub of the body's cellular immunity and humoral immunity. However, the effects of ER on splenic lymphocytes have not been reported. We will further study the mechanism of action of ER on splenic lymphocytes.

## 5 Conclusions

Overall, the results of this study indicated that ER significantly ameliorated the symptoms of immune and digestive disorder symptoms in RRR-induced spleen deficiency rats. The therapeutic mechanism of ER in spleen deficient rats was closely related to the modulation of intestinal metabolites and intestinal flora as well as the P38MAPK pathway. This study provides new insight into the role of ER as a unique potential prebiotic, demonstrating its therapeutic effects from the perspective of the intestinal metabolites and intestinal flora, which may help develop health products and provide more effective treatment strategies for spleen deficiency syndrome.

## Data Availability

The original contributions presented in the study are included in the article/supplementary material, further inquiries can be directed to the corresponding author.
